# A Novel Method of Placing Right Ventricular Leads in Patients With Persistent Left Superior Vena Cava Using a Conventional J Stylet

**DOI:** 10.1016/s0972-6292(16)30731-8

**Published:** 2014-03-12

**Authors:** Guillermo Mora

**Affiliations:** Associate Professor, Universidad Nacional de Colombia

**Keywords:** persistent left superior vena cava, pacemaker, implantation

## Abstract

**Background:**

Locating pacemaker electrodes can become complicated by congenital abnormalities such as persistent left superior vena cava (LSVC).

**Objective:**

To evaluate a technique for the implanting of ventricular electrode in patients with persistent LSVC.

**Materials and Methods:**

The study was carried out from June 2001 to June 2010 involving all patients who were admitted to the Hospital Universitario Mayor, Instituto de Corazon de Bogota and Hospital Universitario Clinica San Rafael (Bogota-Colombia) for implanting pacemakers or cardiac defibrillators. LSVC was diagnosed by fluoroscopic observation (anterior-posterior view) of the course of the stylet. Four steps were followed: 1) Move the electrode with a straight stylet to the right atrium. 2) Change the straight stylet by a conventional J stylet and push the electrode to the lateral or anterolateral wall of the right atrium. 3) Remove the guide 3-5 cm and 4) Push the electrode which crosses the tricuspid valve into the right ventricle and finally deploy the active fixation mechanism.

**Results:**

A total of 1198 patients were admitted for pacemaker or cardiac defibrillator implant during the 9-year study period, 1114 received a left subclavian venous approach. There were 573 males and 541 females. Persistent LSVC was found in five patients (0.45%) Fluoroscopy time for implanting the ventricular electrode ranged from 60 to 250 seconds, 40 to 92 minutes being taken to complete the whole procedure.

**Conclusion:**

We present a simple and rapid technique for electrode placement in patients with LSVC using usual J guide and active fixation electrodes with high success.

## Introduction

Locating electrodes can become complicated by congenital abnormalities including alterations of the systemic upper veins, such as persistent left superior vena cava (LSVC). Such alteration may involve the right superior vena cava (RSVC) (double system) or just LSVC (20% of cases) [1]. The absence of superior vena cava drainage may even be found in some patients [2]. Persistent LSVC has been reported in autopsy studies in around 0.3% of the general population [3], but may increase to 3%–10% in patients suffering other congenital abnormalities [4-6]. The prevalence of LSVC may be underestimated as a certain amount of pacemaker and defibrillator implants are performed on the right side as first choice [7]. However, Biffi et al., found 0.41% prevalence with the absence of RSVC in 36% of cases in this population [8].

This work presents a technique for gaining access to the right ventricle using a conventional J stylet and active fixation electrodes.

## Materials and Methods

The study was carried out from June 2001 to June 2010 involving all patients who were admitted to the Hospital Universitario Mayor, Instituto de Corazon de Bogota and Hospital Universitario Clinica San Rafael (Bogota-Colombia) to have a pacemaker or cardiac defibrillator implanted. This did not include patients attending for their generators to be changed.

The technique of subclavian vein access was direct puncture. Persistent LSVC was diagnosed by fluoroscopic observation (anterior-posterior view) of the course of the stylet that it entered the subclavian vein and then descended parallel to the spine without crossing it to the right; it then took the course of the coronary sinus and passed the spine, being observed afterwards in the right atrium (RA). Other signs which help the diagnosis are: left paravertebral shadow above aortic bow and no shadow at the right in case of absence of RSVC.

**Figure 1:**
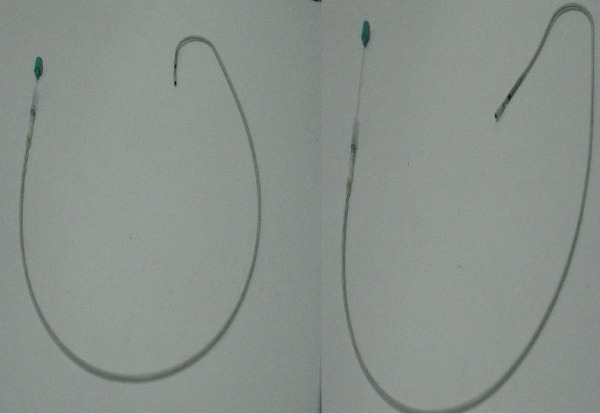
Movement of the electrode tip when removing the J stylet

**Figure 2:**
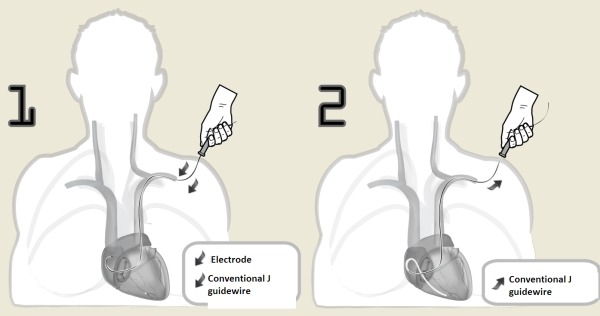
Electrode with J stylet inside carried right atrium until the tip is placed opposite the tricuspid valve (1) and while we maintain the fixed electrode, the J stylet is removed 3 cm, we observe the movement of the electrode tip into the right ventricle (2).

**Figure 3:**
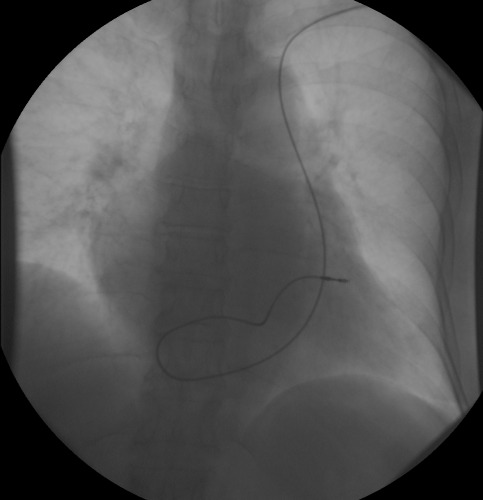
Defibrillator electrode implanted in the right ventricular apex.

**Figure 4:**
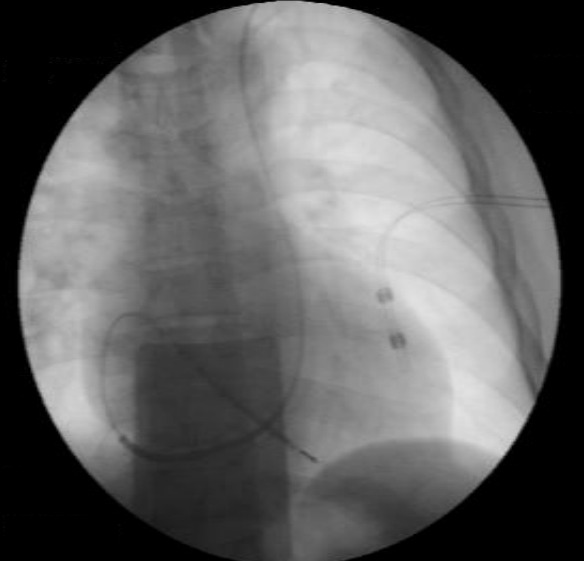
Pacemaker electrode implanted in the right ventricular septum

The following technique was used for gaining access to the right ventricle (RV) in patients diagnosed with persistent LSVC. Four steps were carried; in the first, the electrode was initially introduced with a straight stylet as far as the RA. The second step was to change the straight stylet for a conventional J stylet and the electrode was pushed towards the lateral or anterolateral wall of the RA. The electrode tip was thus lying against the tricuspid valve. It is important to emphasize that we use the standard J guide that comes in all implant kits and is not a stylet for the implanters to do with their hands. The third step is, in this position, to remove the stylet 3-5 cm. This is the most important step, since with the withdrawal of the stylet, without moving the electrode, the electrode tip is directed forwards (Figure 1), as it was previously facing the tricuspid annulus it passes easily through the tricuspid valve (Figure 2,3). To remove the J stylet 3-5 centimeters, the tip of the guide pushes the electrode up in the contact site and the electrode tip is directed forward. The last step is to push the electrode to make contact with the ventricular endocardium and finally to deploy the active fixation mechanism. If it were wished to leave it in the apex, then an clockwise rotation would have been needed before withdrawing the guide; on the contrary, if it were wished to place it in the septum or in the outflow tract, then it would have had to be a counterclockwise rotation (Figure 3,4). Once the endocardial surface was reached, the active fixation mechanism was used and stimulation and sensed parameters were checked. If the parameters were not seen to be suitable, then the technique had to be repeated with slight changes in clockwise or anticlockwise rotation.

## Results

A total of 1198 patients were admitted for pacemaker or cardiac defibrillator implants during the 9-year study period and 1114 received a left subclavian venous approach. There were 573 males and 541 females. Persistent LSVC was found in five patients (0.45%). A left subclavian approach could not be made in 84 of these patients, as access could not be gained to the vein (puncture failure) in 30 of them. Other causes were seen in the rest of the patients, such as having a background of breast cancer with radiotherapy or surgery, having left subclavian access for temporary pacemakers or central catheters, or the presence of cutaneous lesions (e.g. severe allergic dermatitis) in the puncture area.

### Description of cases

The first case was a male patient aged 76 who had a consultation for a syncopal event documented in Holter monitoring as being atrioventricular (AV) block Mobitz type II. Echocardiogram was normal. A VVIR pacemaker was successfully implanted in the patient, followed by 3 years with suitable stimulation and sensed parameters. The patient died from lung cancer. The second patient was a 72 year-old male who was admitted for presyncopal events documented as being complete AV block. Echocardiogram was normal. A VVIR pacemaker was successfully implanted and he has been followed up for 5 years with good functioning parameters.

The third patient was a 73 year-old female who was admitted for complete AV block with syncope. Echocardiogram was normal. A VVIR pacemaker was implanted which has been followed up for 2 years with suitable functioning parameters.

The fourth patient was a 64 year-old male suffering from non-ischemic dilated cardiopathy (15% left ventricular ejection fraction), functional class II and suitable management of cardiac failure; his serum proved negative for antibodies against Chagas' disease. He was admitted for a single-chamber cardiac defibrillator implant for primary prevention of sudden death. The device was successfully implanted with suitable stimulation and sensed parameters and a defibrillation threshold of less than 15 joules. The defibrillation shock was programmed to occur between the generator body and the electrode tip. The patient has been followed up for 4 years without changes in its parameters.

The last patient is a 63 year old man with presíncope events and findings of sick sinus syndrome. Echocardiogram was normal. A dual chamber pacemaker implanted. This patient has been followed for 6 months without changes in its parameters.

Characteristics of the stimulation and sensing parameters at implant are presented in Table 1. At follow-up no changes in these parameters. Fluoroscopy time for implanting the ventricular electrode ranged from 1 to 4 minutes, 40 to 92 minutes being taken to complete the whole procedure. In these cases, we used J stylets of St Jude Medical and Boston Scientific. The first patient had LSVC attempts to place the ventricular with a U-shaped stylus, but we were unable to do so, after 5 minutes it was attempted with the guidawire in the form of J. The procedure lasted 4 minutes and needed to be repositioned due to poor pacing thresholds. In this case the procedure was repeated with a counterclockwise rotation. In all cases, we used electrodes of 58 cm and we had no problems with the length to reach the ventricular endocardium. None of the patients evaluated had complications in the short or long term.

## Discussion

The finding of persistent LSVC when fitting a pacemaker or cardiac defibrillator is considered to be an event which complicates such procedures because the tip of the lead is deflected away from the tricuspid orifice.

The embryological development of the superior vena cava is complex. In a 4mm (week 4) embryo, the principal vein formation that can be distinguished is the sinus vein, where three vein groups drain [9]. The vitelline vein system transports blood from the vitelline sac; and the umbilical vein system brings blood from the placenta and the cardinal vein system, which is completely intraembryonary. The anterior and posterior cardinal veins drain to the right and left of the venous sinus. The common cardinal veins begin at the point where they join. At 15–17mm, the right umbilical vein disappears and the left umbilical vein connects distal to the hepatic plexus (venous conduit). The left vitelline vein atrophies and the right vitelline vein contributes to form the inferior vena cava. A bridge develops between both cardinal veins after about 8 weeks via the innominate vein [10]. The common right cardinal will ultimately become the RSVC and the common left cardinal will atrophy leaving only a small channel, the coronary sinus. If this does not atrophy, we call this persistent LSVC draining in the coronary sinus. In 92% of cases, drainage occurs in the right atrium; in the remainder of cases, drainage occurs in the left atrium because of failure to form the coronary sinus [11].

Several LSVC subtypes have been described. There is no RSVC in the first subtype and all venous drainage from the head and both arms is pumped to the coronary sinus and the RA via the LSVC. The LSVC and RSVC exist in the second subtype, ipsilaterally draining the venous system of the head and upper members; the RSVC drains to the RA and the LSVC to the coronary sinus. An innominate vein persists in a third subtype which joins the LSVC and the RSVC; each is drained as in the previous subtype. Any of the previously described alterations may be present in a last subtype, in which the LSVC drains into the left atrium.

The pacemaking tissue of the heart is derived from two sites near the progenitors of the superior vena cava. The right-sided site forms the sinoatrial node; the left-sided site is normally carried down to an area near the coronary sinus. Anomalies of the coronary sinus may be associated with abnormalities of the conduction system of the heart. This may be due to the close proximity of the coronary sinus to the final position of the left-sided primitive pacemaking tissue. It has been proposed that there is a relationship between such anomaly and conduction defects caused by stretching of the AV node and the His bundle; hypoplasia of the sinus node and the AV node have also been reported [12]. These structures` development seems to be related to the development of the venous system, since they are located in the connection of the right and left cardinal veins with the sinus venosus. An alteration in the location and histologic organization of the sinus node and AV node have also been described and even alterations in arterial suplency [13-15]. Abnormal persistent fetal dispersion of specialized pacemaker and conduction tissue, which occurs in some individuals with persistent LSVC, may provide an arrhythmogenic substrate [16]. Its incidence in congenital heart disease varies (2–5%) and it is more frequent in stenosis or pulmonary atresia, D-transposition, complete atrioventricular septal defects, anomalous pulmonary vein drainage, and cor triatriatum [17-20].

Trans-venous positioning of a pacemaker or ICD lead through persistent LSVC may be technically challenging, particularly in the absence of a bridging innominate vein between the persistent LSVC and right superior vena cava. Common techniques of implantation via persistent LSVC consist of pre-shaping the stylets in different angles in order to perform a U-turn of the electrode from the coronary sinus to the right atrium and into the right ventricle. Previous approaches include: (a) manual reshaping and sizing of the stylet into a U-shaped stylet, necessitating considerable manoeuvring, depending on the right heart size and geometry. (b) Forming a loop in the right atrium using right atrial free wall for support. (c) Utilizing of atrial J lead for ventricular lead placement. (d) Ventricular lead placement in the left ventricular branch of the coronary sinus.

Most techniques imply deforming a stylet into an U- or L-shape for directing the tip of the electrode to the tricuspid ring [1,21]. Approaches such as curving the stylet into a pigtail [22], using an L-shaped lead [14] or a wide loop have been described [23]. Dirix et al [24] used a pre-shaped J-lead, after manipulation in a posteroanterior plane, the tricuspid valve could easily be passed. However, the whole procedure was completed within 3 hours. Although initially anyone can believe that this technique is similar to ours, electrode movement of our technique is done without the J stylet at the tip, whereby the electrode does not pass the tricuspid valve shaped J. Using the preformed atrial electrode, manipulation in the ventricle may be difficult and the use of atrial electrodes in some patients can be too short to reach the right ventricle. Gaba et al [25] placed a ventricular lead via a coronary vein with an over-the-wire system and achieve long-term stability and acceptable chronic pacing thresholds.

Other authors have used different devices are not usually found in the electrophysiology laboratory. Fukuda et al [26] used a steerable stylet (St Jude) for insertion of pacing leads into not only the right atrium but also the right ventricle. Daccarett et al used a special sheath (a 40 cm 9 F CSG Worleyw) and within 2 min after sheath deployment, the lead was positioned in the right ventricle apex without any complications [27].

The technique described with four steps has the advantage of not needing to preform a stylet (that will change with each operator), or the use of special equipment, but rather using a standard J stylet, which is found in all pacemaker electrode package. In these cases Boston and St Jude stylets were used. It needs little manipulation since it only has to be given a slight clockwise or anticlockwise rotation on withdrawing the stylet, has a high success rate (100% in our series) and is accompanied by less fluoroscope time and procedure than other series [1] and case reports [21-23] in the pertinent literature. Electrode dysfunction has not been found in patient follow-ups. We strongly recommend the use of active fixation systems, others have reported frequent electrode dislocation [28-30] which will happen less frequently by using active fixation systems [31,32].

Unless specifically searched for, the presence of a persistent LSVC may be overlooked, including on echocardiography. Therefore, implanting physicians are usually presented with an unexpected finding that requires the use of tools not readily available in the electrophysiology laboratory [33]. A suitable and easily-managed technique must be available for this group of patients, as lack RSVC (not evaluated in this series) as this may be the only way of providing an endocardial approach to locating an electrode.

## Limitations

The technique described here corresponds to the experience of a single operator and the same success may not be achieved in other hands; however, given the easiness of the manoeuvre, it is very probable that all operators could use it with equal effectiveness.

On the other hand, it needs the use of active fixation electrodes, above all for stability in septal localisation or the outflow tract; however, active fixation systems are required at these sites (even in patients presenting no anomalies) for reducing the risk of displacement.

Although the global trend is to use dual-chamber pacemakers, we only use unicameral pacemaker in our patients with AV block. One study found no substantial benefit with the use of DDD vs VVI pacemakers in elderly patients with AV block [34]. Our patients were elderly and our health care system, of developing country, has limited resources.

Although we were able to place the ventricular lead in all patients, it is possible that very large right atriums need longer electrodes than those used in this study. Finally, our study did not compare different techniques, since in this patients` tricuspid valve passes it is difficult and repeat the procedure with another technique (with display the active fixation mechanism) can present risks to patients.

## Conclusions

A technique has thus been presented for placing an electrode in the right ventricle of patients suffering from LSVC persistence using a conventional J guide and active fixation electrodes. The J wire is a tool that could be helpful in these patients.

## Figures and Tables

**Table 1:**
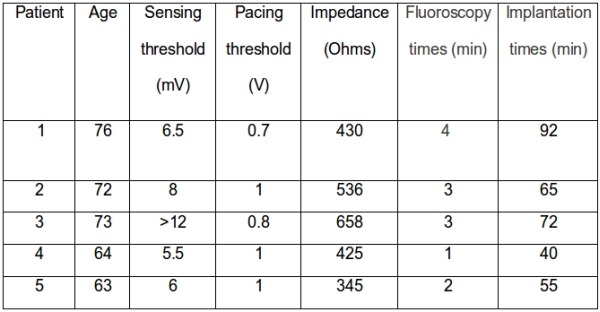
Characteristics of the stimulation and sensing parameters at implant
